# Relationship Between Serum Nell‐1 Level and Bone Geometry, Bone Microarchitecture in Chinese Postmenopausal Women

**DOI:** 10.1155/ije/9977862

**Published:** 2026-01-31

**Authors:** Yiyi Gong, Yushuo Wu, Xiang Li, Xiaosen Ma, Xiaolin Ni, Wei Liu, Lijia Cui, Yue Chi, Ruizhi Jiajue, Qianqian Pang, Ou Wang, Mei Li, Xiaoping Xing, Zaizhu Zhang, Wei Yu, Yan Jiang, Weibo Xia

**Affiliations:** ^1^ Department of Endocrinology, Key Laboratory of Endocrinology, National Commission of Health, Peking Union Medical College Hospital, Chinese Academy of Medical Science, No. 1 Shuaifuyuan Wangfujing Street Dongcheng District, Beijing, 100730, China, cams.ac.cn; ^2^ Center for Biomarker Discovery and Validation, National Infrastructures for Translational Medicine, Institute of Clinical Medicine, Peking Union Medical College Hospital, Chinese Academy of Medical Sciences, No. 1 Shuaifuyuan Wangfujing Street Dongcheng District, Beijing, 100730, China, cacms.ac.cn; ^3^ Department of Endocrinology, Beijing Tsinghua Changgung Hospital, School of Clinical Medicine, Tsinghua University, 168 Litang Road Changping District, Beijing, 102218, China, tsinghua.edu.cn; ^4^ Department of Endocrinology, Genetics and Metabolism, Beijing Children’s Hospital, Capital Medical University, National Center for Children’s Health, Beijing, 100045, China, bch.com.cn; ^5^ Department of Radiology, Peking Union Medical College Hospital, Chinese Academy of Medical Sciences and Peking Union Medical College, Beijing, 100730, China, cacms.ac.cn

**Keywords:** bone microarchitecture, bone mineral density, fall risk, muscle, Nell-1, postmenopausal women

## Abstract

**Purpose:**

Neural EGF‐like 1 (Nell‐1), originally implicated in craniosynostosis, has been identified as a key regulator in osteogenic processes. While preclinical data were encouraging, clinical studies correlating serum Nell‐1 levels with osteoporosis remain scarce. This study aims to investigate the relationship between circulation Nell‐1 level and bone turnover markers, bone mineral density (BMD), bone microstructure, muscle strength, fall risk, and fractures in Chinese postmenopausal women.

**Methods:**

Serum Nell‐1 levels were measured in 123 Chinese postmenopausal women. Muscle function was evaluated through grip strength tests, the Short Physical Performance Battery (SPPB), and the Timed Up and Go (TUG) test. Dual‐energy X‐ray absorptiometry was used to assess areal bone mineral density (aBMD), lumbar trabecular bone score (TBS), and muscle mass. High‐resolution peripheral quantitative computed tomography (HR‐pQCT) was applied to determine volumetric bone mineral density (vBMD), analyze bone microarchitecture, and estimate bone strength.

**Result:**

Postmenopausal women with higher serum Nell‐1 levels had higher aBMD and total volumetric bone mineral density (Tot.vBMD) at the distal tibia, larger cortical area (Ct.Ar) and thicker cortical thickness (Ct.Th) at the distal tibia, and higher bone strength. There was a significant negative association between serum Nell‐1 levels and C‐terminal cross‐linking telopeptide of type I collagen (β‐CTX), while no significant correlations were observed between serum Nell‐1 levels and muscle mass or function.

**Conclusion:**

Postmenopausal women with higher serum Nell‐1 levels exhibited higher BMD and bone strength, indicating its potential as a therapeutic invention for osteoporosis.

## 1. Introduction

Osteoporosis (OP), a prevalent skeletal disorder characterized by reduced bone mass, compromised bone microarchitecture and increased bone fragility, poses a significant health challenge, particularly for postmenopausal women. The physiological decline in estrogen levels following menopause accelerates bone loss and muscle deterioration, culminating in an increased risk of falls and fractures [1–3]. According to epidemiological studies conducted in China, the prevalence of OP among postmenopausal women is 32.1% [4], and the age‐standardized prevalence of OP was 29.1% among women aged 50 years or older [5]. These chronic conditions not only augment the risk of fragility and mortality but also significantly impair the quality of life and exacerbate the economic burden for postmenopausal women [6–8]. With the escalating challenge of an aging population, the prevention and management of OP are becoming increasingly critical for safeguarding public health. In this context, the identification and understanding of biomarkers that influence bone health have critical importance.

Neural EGF‐like 1 (Nell‐1), a protein initially associated with craniosynostosis [9], has emerged as a pivotal factor in osteogenic. Over the past two decades, research has expanded our understanding of Nell‐1’s function, particularly in osteogenesis, and bone regeneration [10]. Recent studies have further delineated the influence of Nell‐1 on skeletal development, highlighting its potential as a therapeutic intervention for OP [10, 11]. Previous study identified a significant correlation between genetic variant in the *NELL1* gene (rs10766761) and reduced bone mineral density (BMD), suggesting a genetic link to OP susceptibility [12]. Preclinical studies in aging rats have demonstrated a decrease in Nell‐1 protein expression concomitant with a decline in bone density [13]. Intriguingly, localized administration of recombinant human Nell‐1 (rhNell‐1) effectively enhances bone density in osteoporotic animal models [14].

Despite these promising preclinical results, clinical cohort studies that substantiate the relationship between circulating Nell‐1 levels and bone are limited, particularly regarding its influence on bone density and microarchitecture. Additionally, the potential associations between Nell‐1, muscle mass, fall risk, and fracture susceptibility remain unexplored. Addressing these gaps, this study aims to investigate the correlation between circulating Nell‐1 expression levels and various OP‐related parameters, such as BMD, bone microarchitecture, muscle mass, the risk of falls, and fractures, in a cohort of postmenopausal Chinese women.

## 2. Methods

### 2.1. Subjects

This investigation was a cross‐sectional study among community‐dwelling postmenopausal women based on the Beijing subset of the Chinese Vertebral Osteoporosis Study (ChiVos). All procedures were performed in accordance with the approval of ethical committee of Peking Union Medical College Hospital (JS‐2905). The participants were enrolled between September and December in 2021. The selection criteria encompassed: (1) aged more than 50 years; (2) a residential history within a Beijing urban community for a minimum of six months, and (3) cessation of menstruation for at least 12 months via self‐report or at least 6 months post‐bilateral oophorectomy. The exclusionary parameters were as follows: (1) non‐Asian ethnicity and (2) the presence of cognitive impairment or physical disability. The research protocol received clearance from our center. Each participant was thoroughly informed about the study objectives, procedures, and potential implications, culminating in the acquisition of informed consent.

### 2.2. Clinical Data Collection

By administering a questionnaire, the general information was collected. Fall and fracture histories were reported by participants. Fractures were validated through X‐rays. Anthropometric measurements, height and weight, were precisely taken and subsequently utilized to calculate the body mass index (BMI) for each participant.

### 2.3. Biochemical Measurements

Blood samples were obtained from all participants in a fasting state and centrifuged at 3000 rpm for 10 min. A comprehensive biochemical profile was assessed by automated analyzer (Beckman Coulter AU5800, USA), which included levels of alanine aminotransferase (ALT), fasting blood glucose (FBG), total cholesterol (TC), total triglycerides (TG), low‐density lipoprotein cholesterol (LDL‐C), high‐density lipoprotein cholesterol (HDL‐C), serum calcium, phosphate, alkaline phosphatase (ALP), and creatinine (Cr). Parathyroid hormone (PTH) levels were measured by a separate autoanalyzer (Beckman Coulter DXI800, USA). Further assessments included the quantification of 25‐hydroxyvitamin D [25(OH)D], procollagen type 1 N‐terminal pro‐peptide (P1NP), and C‐terminal cross‐linking telopeptide of type I collagen (β‐CTX), which were conducted by the electro‐chemiluminescence immunoassay on a Roche Cobas E601 analyzer (Roche Diagnostics, Switzerland). The measurement of serum Nell‐1 level was performed using enzyme‐linked immunosorbent assay (ELISA) kits (Cat No. ELH‐NELL1, RayBiotech, USA) according to the manufacturer’s protocol.

### 2.4. Dual‐Energy X‐ray Absorptiometry (DXA) Assessment

In the current study, a bone density measurement device (Lunar, GE Healthcare, Madison, USA) was utilized to evaluate the areal bone mineral density (aBMD), trabecular bone score (TBS), and muscle mass of the participants. By DXA, we conducted scans to determine aBMD at three key sites: the total hip, femoral neck, and lumbar vertebrae 1 through 4 (L1‐4). The raw bone density measurements were normalized into T‐scores by comparing them against the optimal bone mass of a young adult reference population of the same sex and ethnicity, utilizing the Chinese reference database provided by GE Lunar. Classification of bone status was based on the T‐scores, with the following criteria: normal bone mass (T‐score ≥ −1.0), osteopenia (−2.5 < T‐score < −1.0). OP was diagnosed when the T‐score of the bones was ≤ −2.5, or in the presence of fragility fractures affecting the hip or vertebrae, and also considered for individuals with low bone mass who had a history of fragility fractures in the proximal humerus, pelvis, or distal forearm. The %CV of BMD measurements at LS, FN, and TH was 0.9%, 1.8%, and 0.7%, respectively; the LSC of BMD measurements at LS, FN, and TH was 2.493%, 4.986%, and 1.939%, respectively.

As with aBMD, TBS collected data using similar process, but different algorithms and analysis software was used. Using the TBS iNsight software (Medimaps), TBS values at the lumbar spine were calculated. In parallel, body composition was evaluated using the enCORE (version 10.50.086) to ascertain the appendicular skeletal muscle mass (ASM) and the appendicular skeletal muscle mass index (ASMI). The diagnosis of sarcopenia is based on the guidelines established by the Asian Working Group for Sarcopenia (AWGS) in 2019 consensus statement [15].

### 2.5. High‐Resolution Peripheral Quantitative CT (HR‐pQCT) Analysis

In accordance with the established protocol, HR‐pQCT scans were performed using the XtremeCT II scanner (Scanco Medical, Brüttisellen, Switzerland) [16]. A comprehensive set of HR‐pQCT parameters was assessed, including the total area (Tot.Ar), trabecular area (Tb.Ar), cortical area (Ct.Ar), cortical perimeter (Ct.Pm), total volumetric bone mineral density (Tot.vBMD), trabecular volumetric bone mineral density (Tb.vBMD), cortical volumetric bone mineral density (Ct.vBMD), trabecular number (Tb.N), trabecular thickness (Tb.Th), trabecular separation (Tb.Sp), cortical thickness (Ct.Th), and cortical porosity (Ct.Po). The bone strength was further estimated through the computation of stiffness and failure load, utilizing the Scanco Finite Element Analysis software (version 1.13) according to the preset automated workflow.

### 2.6. Physical Performance Evaluation

In accordance with the 2019 guidelines established by the AWGS, female participants were identified as having sarcopenia if they presented with an ASMI below the threshold of 5.4 kg/m^2^, along with diminished grip strength of less than 18 kg. Additional criteria for sarcopenia classification included a reduced physical performance as indicated by a Short Physical Performance Battery (SPPB) test [17] and the Timed Up and Go (TUG) test [18].

### 2.7. Statistics

Statistical analyses were conducted using SPSS software (version 25.0). The Kolmogorov–Smirnov test was used to assess the distribution of continuous variables. Variables exhibiting a normal distribution were expressed as mean ± standard deviation (SD), while those with non‐normal distribution were presented as median (interquartile range) (IQR). Categorical data were reported as proportion (counts/sum). For comparing variables across multiple groups, one‐way analysis of variance (ANOVA) and Kruskal–Wallis H test were selected based on the data distribution. *p* values were adjusted using the Bonferroni correction method to control for Type I error. Chi‐square or Fisher’s exact test was utilized for comparing categorical data among groups. Unadjusted bivariate correlations were investigated using Spearman and Kendall’s tau‐b correlation analyses. The Mann–Whitney *U* test was employed to evaluate differences between two groups. Statistical significance was set at a threshold of *p* < 0.05.

## 3. Results

### 3.1. Baseline Characteristics of the Participants

A total of 123 postmenopausal women with serum Nell‐1 level results were included. The demographic and biochemical profiles of the participants are delineated in Table 1. The median age of the participants was 69.0 years old (IQR, 64.0–78.0 years). The median serum Nell‐1 level was 0.155 (0.095, 0.253). The mean serum calcium (Ca) level was 2.35 ± 0.09 mmol/L. The serum levels of phosphorus (Pi), ALP, intact parathyroid hormone (iPTH), 25‐hydroxyvitamin D (25[OH]D), C‐terminal telopeptide of type I collagen (β‐CTX), and N‐terminal pro‐peptide of type I collagen (P1NP) were 1.18 (1.07, 1.31) mmol/L, 79.0 (71.0, 91.0) U/L, 48.6 (36.8, 63.4) pg/mL, 21.6 (16.4, 27.1) ng/mL, 0.40 (0.28, 0.49) ng/mL, and 45.7 (35.0, 57.7) ng/mL, respectively.

**TABLE 1:** Baseline characteristics of the cohort.

	Overall (*N* = 123)	T1 (*n* = 41)	T2 (*n* = 41)	T3 (*n* = 41)	*p*
General characteristics					
Age (y)	69.0 (64.0, 78.0)	73.0 (69.0, 80.5)	69.0 (66.5, 75.0)	69.0 (64.0, 77.0)	0.212
Height (cm)	155.3 ± 6.8	153.9 ± 6.5	155.0 ± 7.9	157.1 ± 5.5	0.092
Weight (kg)	60.0 (54.0, 68.0)	60.0 (54.0, 67.5)	59.5 (50.0, 65.8)	63.0 (58.0, 70.5)	0.057
BMI (kg/m^2^)	25.57 ± 4.05	25.98 ± 4.15	24.50 ± 3.66	26.21 ± 4.20	0.117
Menopausal age (y)^a^	50.0 (47.0, 52.0)	50.0 (48.0, 52.0)	48.5 (47.0, 51.5)	50.0 (47.0, 52.0)	0.346
Biochemical parameters					
Nell‐1 (ng/mL)	**0.155 (0.095, 0.253)**	**0.072 (0.011, 0.095)**	**0.155 (0.125, 0.173)**	**0.304 (0.253, 0.567)**	**< 0.001**
Ca (mmol/L)	2.35 ± 0.09	2.36 ± 0.15	2.35 ± 0.09	2.35 ± 0.11	0.924
Pi (mmol/L)	1.18 (1.07, 1.31)	1.18 (1.09, 1.31)	1.24 (1.08, 1.36)	1.17 (1.05, 1.28)	0.294
ALP (U/L)	79.0 (71.0, 91.0)	80.0 (68.0, 95.0)	80.0 (70.0, 95.0)	78.0 (71.5, 90.0)	0.711
iPTH (pg/mL)	48.6 (36.8, 63.4)	48.5 (37.3, 59.0)	50.3 (35.7, 62.4)	49.9 (37.3, 65.6)	0.715
25(OH)D (ng/mL)	21.6 (16.4, 27.1)	21.3 (17.1, 29.4)	18.7 (13.8, 26.6)	22.6 (17.2, 26.5)	0.191
β‐CTX (ng/mL)	0.40 (0.28, 0.49)	0.42 (0.31, 0.52)	0.41 (0.27, 0.58)	0.37 (0.21, 0.47)	0.138
P1NP (ng/mL)	45.7 (35.0, 57.7)	49.3 (34.7, 64.4)	48.7 (35.3, 61.7)	42.4 (34.7, 55.5)	0.383
Cr (μmol/L)	64.0 (58.0, 77.0)	69.0 (59.5, 84.0)	63.0 (56.5, 70.0)	64.0 (58.0, 73.5)	0.048
Medication history					
Used/using glucocorticoid (≥ 3 months)^b^	4.2% (5/120)	5.0% (2/40)	5.0% (2/40)	2.5% (1/40)	0.812
VitD and calcium supplementation (≥ 3 months)	32.5% (40/123)	24.4% (10/41)	41.5% (17/41)	31.7% (13/41)	0.254
HRT (≥ 3 months)^a^	5.8% (7/121)	7.5% (3/40)	5.0% (2/40)	4.9% (2/41)	0.851
Anti‐osteoporotic drugs (≥ 3 months)^a^	14.9% (18/121)	15% (6/40)	17.5% (7/40)	12.2% (5/41)	0.798
Percentage of osteoporosis and sarcopenia					
Osteoporosis^a^	24% (29/121)	20% (8/40)	25.0% (10/40)	26.8% (11/41)	0.758
Sarcopenia	3.3% (4/123)	0% (0/41)	7.3% (3/41)	2.4% (1/41)	0.164

*Note:* All subjects were grouped according to tertiles of serum Nell‐1 level. Normally distributed continuous variables were depicted as mean ± standard deviation (SD) and non‐normally distributed continuous variables were shown as median (interquartile range, IQR). Categorical variables were expressed as proportion (counts/sum). Normally distributed continuous variables were analyzed by the one‐way analysis of variance (ANOVA). Non‐normally distributed continuous variables were analyzed by the Kruskal–Wallis H test. The classified data was analyzed by the Chi‐square test or Fisher’s exact test. Bold values denote statistically significant differences (*p* < 0.05). Ca, serum total calcium; Pi, serum phosphate; ALP, serum alkaline phosphatase; iPTH, serum intact parathyroid hormone; 25(OH)D, 25‐hydroxy vitamin D; β‐CTX, C‐terminal cross‐linking telopeptide of type I collagen; P1NP, procollagen type 1 N‐terminal pro‐peptide; Cr, serum creatinine; VitD, vitamin D.

Abbreviations: BMI, body mass index; HRT, hormone replacement therapy.

^a^ *N* = 121, T1 *n* = 40, T2 *n* = 40, T3 *n* = 41.

^b^ *N* = 120, T1 *n* = 40, T2 *n* = 40, T3 *n* = 40.

Among the participants, 29 (24.0%) were diagnosed with OP, and approximately 3.3% were diagnosed with sarcopenia. Participants were divided into three groups based on tertiles of serum Nell‐1 level, and no significant differences were found in general characteristics and biochemical parameters between each tertile. Additionally, we conducted association analysis between the serum Nell‐1 level and general characteristics, also with biochemical parameters (Supporting Table 1). There was a positive correlation between serum Nell‐1 level and height (*r* = 0.206, *p* = 0.022). Negative correlation was observed between Nell‐1and β‐CTX (*r* = −0.183, *p* = 0.043).

### 3.2. Association Between Serum Nell‐1 Level and aBMD, Lumbar TBS, and HR‐pQCT Parameters

The association between serum Nell‐1 level and aBMD, and lumbar TBS are shown in Table 2 and Supporting Table 2. There was a statistically significant difference in the aBMD of the femoral neck among the three groups (*p* = 0.015). Further analysis among groups, the aBMD of the femoral neck was highest in the highest tertile group of serum Nell‐1 level, with a statistically significant difference compared to the second tertile group of serum Nell‐1 level (*p* = 0.015). T score of the aBMD of the femoral neck had the same results and *p* value among three groups was 0.016. After trisection stratification comparison, the *p* value between the highest tertile group and the second tertile group was still < 0.05 (*p* = 0.013). A similar trend was observed for total hip aBMD and the T‐score of the aBMD. Three groups had significantly different total hip aBMD (*p* = 0.019). The highest tertile group of serum Nell‐1 level had the highest total hip aBMD, showing a statistically significant difference when compared to the second tertile group of serum Nell‐1 levels (*p* = 0.015). The T‐score of the aBMD had a *p* value of 0.019 when comparing the three groups. Further analysis revealed that only the difference between the highest and second tertile groups remained statistically significant (*p* = 0.016). No significant differences in aBMD of L1‐4 and lumbar TBS across different tertiles of serum Nell‐1 level. In bivariate correlation analysis, no correlation was found in serum Nell‐1 level and aBMD, also lumbar TBS.

**TABLE 2:** aBMD, lumbar TBS, and HR‐pQCT parameters among postmenopausal women according to tertiles of serum Nell‐1 level.

	ALL (*N* = 123)	T1 (*n* = 41)	T2 (*n* = 41)	T3 (*n* = 41)	*P*	*P*_1_	*P*_2_	*P*_3_
aBMD								
L1‐4	1.056 ± 0.183	1.060 ± 0.194	1.023 ± 0.184	1.083 ± 0.169	0.328	1.000	1.000	0.416
L1‐4T	−0.56 ± 1.48	−0.51 ± 1.57	−0.83 ± 1.50	−0.33 ± 1.35	0.303	0.991	1.000	0.382
Femoral neck	**0.774 ± 0.129**	**0.762 ± 0.123**	**0.740 ± 0.126**	**0.819 ± 0.128**	**0.015**	1.000	0.127	**0.015**
Femoral neck T‐score	−**1.16 ± 1.08**	−**1.22 ± 1.02**	−**1.46 ± 1.06**	−**0.80 ± 1.06**	**0.016**	0.867	0.207	**0.013**
Total hip	**0.843 ± 0.146**	**0.848 ± 0.137**	**0.796 ± 0.155**	**0.886 ± 0.135**	**0.019**	0.306	0.688	**0.015**
Total hip T‐score	−**0.89 ± 1.17**	−**0.86 ± 1.10**	−**1.27 ± 1.24**	−**0.55 ± 1.07**	**0.019**	0.320	0.675	**0.016**
lumbar TBS^a^	1.260 ± 0.096	1.252 ± 0.081	1.263 ± 0.110	1.265 ± 0.096	0.806	1.000	1.000	1.000
HR‐pQCT parameters of radius								
Bone geometry								
Tot.Ar (mm^2^)	248.5 ± 39.6	246.1 ± 43.2	251.3 ± 37.8	248.2 ± 38.3	0.842	1.000	1.000	1.000
Tb.Ar (mm^2^)	199.7 ± 40.6	198.3 ± 41.8	204.2 ± 38.7	196.5 ± 41.8	0.670	1.000	1.000	1.000
Ct.Ar (mm^2^)	52.3 ± 10.4	51.1 ± 10.6	50.5 ± 9.8	55.1 ± 10.5	0.095	1.000	0.246	0.139
Ct.Pm (mm)	65.9 ± 6.1	65.6 ± 6.6	66.2 ± 5.8	65.8 ± 6.1	0.902	1.000	1.000	1.000
vBMD								
Tot.vBMD (mgHA/cm^3^)	256.8 (211.9, 301.6)	259.6 (215.2, 301.4)	230.7 (192.8, 291.3)	257.4 (229.8, 347.7)	0.144			
Tb.vBMD (mgHA/cm^3^)	91.5 (71.7, 122.6)	101.2 (70.8, 121.6)	84.3 (67.8, 94.7)	105.0 (74.8, 130.9)	0.073			
Ct.vBMD (mgHA/cm^3^)	892.5 ± 70.5	880.5 ± 83.1	894.0 ± 65.6	902.9 ± 60.8	0.355	1.000	0.461	1.000
Bone microarchitecture								
Tb.N (1/mm)	1.123 ± 0.306	1.131 ± 0.285	1.060 ± 0.280	1.178 ± 0.343	0.211	0.883	1.000	0.240
Tb.Th (mm)	0.218 (0.210, 0.229)	0.221 (0.214, 0.231)	0.213 (0.206, 0.227)	0.218 (0.208, 0.231)	0.174			
Tb.Sp (mm)	0.862 (0.737, 1.096)	0.869 (0.729, 1.104)	0.897 (0.804, 1.167)	0.778 (0.704, 1.073)	0.147			
Ct.Th (mm)	0.950 (0.794, 1.049)	0.947 (0.770, 1.066)	0.866 (0.750, 1.049)	0.994 (0.883, 1.087)	0.195			
Ct.Po	0.007 (0.004, 0.010)	0.007 (0.005, 0.010)	0.007 (0.004, 0.009)	0.008 (0.004, 0.011)	0.397			
Estimated bone strength								
Stiffness (N/mm)	**44894.9 ± 11542.9**	**44927.9 ± 11244.2**	**41370.3 ± 11031.9**	**48386.4 ± 111533.1**	**0.021**	0.467	0.502	**0.017**
Failure load (N)	**2386.6 ± 645.2**	**2404.2 ± 621.6**	**2183.3 ± 619.0**	**2572.4 ± 649.6**	**0.022**	0.345	0.688	**0.018**
HR‐pQCT parameters of tibia								
Bone geometry								
Tot.Ar (mm^2^)	651.5 ± 112.5	635.5 ± 112.5	660.6 ± 132.8	658.5 ± 89.0	0.537	0.949	1.000	1.000
Tb.Ar (mm^2^)	559.5 ± 108.9	540.9 ± 111.8	582.7 ± 114.7	554.8 ± 98.1	0.210	0.249	1.000	0.740
Ct.Ar (mm^2^)	**100.7 ± 19.9**	**99.7 ± 18.2**	**93.5 ± 17.0**	**108.9 ± 21.6**	**0.002**	0.427	0.092	**0.001**
Ct.Pm (mm)	99.6 ± 8.2	98.1 ± 8.8	100.7 ± 8.7	99.9 ± 7.0	0.341	0457	0.980	1.000
vBMD								
Tot.vBMD (mgHA/cm^3^)	**221.9 (187.9, 268.2)**	**221.5 (197.3, 267.8)**	**197.9 (175.6, 235.5)**	**238.4 (193.6, 291.2)**	**0.015**			
Tb.vBMD (mgHA/cm^3^)	116.3 ± 38.4	121.2 ± 35.6	104.5 ± 34.6	123.1 ± 42.7	0.053	0.141	1.000	0.083
Ct.vBMD (mgHA/cm^3^)	842.3 ± 65.1	833.0 ± 68.6	842.6 ± 60.0	851.3 ± 66.8	0.449	1.000	0.620	1.000
Bone microarchitecture								
Tb.N (1/mm)	1.125 (0.972, 1.249)	1.083 (0.910, 1.285)	1.115 (0.981, 1.205)	1.154 (1.032, 1.264)	0.273			
Tb.Th (mm)	**0.246 ± 0.020**	**0.254 ± 0.022**	**0.240 ± 0.019**	**0.244 ± 0.016**	**0.003**	**0.003**	0.052	0.972
Tb.Sp (mm)	0.872 (0.783, 1.038)	0.913 (0.770, 1.119)	0.877 (0.818, 1.028)	0.837 (0.771, 0.968)	0.185			
Ct.Th (mm)	**1.216 ± 0.258**	**1.234 ± 0.220**	**1.113 ± 0.238**	**1.301 ± 0.283**	**0.003**	0.086	0.673	**0.002**
Ct.Po	0.033 (0.023, 0.044)	0.037 (0.024, 0.046)	0.032 (0.019, 0.044)	0.032 (0.026, 0.043)	0.313			
Estimated bone strength								
Stiffness (N/mm)	**130107.9 ± 30484.5**	**133147.1 ± 29432.9**	**118490.0 ± 27082.3**	**138901.1 ± 31804.7**	**0.007**	0.079	1.000	**0.007**
Failure load (N)	**7123.2 ± 1625.7**	**7265.3 ± 1569.9**	**6512.5 ± 1449.2**	**7603.6 ± 1693.6**	**0.007**	0.097	1.000	**0.007**

*Note:* All subjects were grouped according to tertiles of serum Nell‐1 level. Normally distributed continuous variables were depicted as mean ± standard deviation (SD) and non‐normally distributed continuous variables were shown as median (interquartile range, IQR). Categorical variables were expressed as proportion (counts/sum). Normally distributed continuous variables were analyzed by the one‐way analysis of variance (ANOVA). Non‐normally distributed continuous variables were analyzed by the Kruskal–Wallis H test. The classified data was analyzed by the Chi‐square test or Fisher’s exact test. *p* values were adjusted using the Bonferroni correction method to control for Type I error. The original significance level was 0.05, and the adjusted significance level was 0.05/3 ≈ 0.0167. *P*_1_ represents the comparison between the T1 and T2 groups; *P*_2_ represents the comparison between the T1 and T3groups; *P*_3_ represents the comparison between the T2 and T3 groups. Bold values denote statistically significant differences (*p* < 0.05, *P*_1_ *P*_2_ *P*_3_ < 0.0167). L1‐4, lumbar vertebrae 1‐4; Tot.Ar, total area; Tb.Ar, trabecular area; Ct.Ar, cortical area; Ct.Pm, cortical perimeter; Tot.vBMD, total vBMD; Tb.vBMD, trabecular vBMD; Ct.vBMD, cortical vBMD; Tb.N, trabecular number; Tb.Th, trabecular thickness; Tb.Sp, trabecular separation; Ct.Th, cortical thickness; Ct.Po, cortical porosity.

Abbreviations: aBMD, areal bone mineral density; HR‐pQCT, high‐resolution peripheral quantitative computed tomography; TBS, trabecular bone score; vBMD, volumetric bone mineral density.

^a^ *N* = 120, T1 *n* = 39, T2 *n* = 40, T3 *n* = 41.

Regarding HR‐pQCT parameters, significant differences were observed in both stiffness and failure load at the distal radius and tibia among the three study groups. Specifically, for the distal radius, the stiffness and failure load exhibited statistically significant differences with *p*‐values of 0.021 and 0.022, respectively. Similarly, at the tibial site, the stiffness and failure load also demonstrated significant disparities with corresponding *p*‐values of 0.007 for each parameter. Further intergroup analysis, the stiffness and failure load were significantly higher in the highest tertile group than the second tertile group (radius:stiffness: *p* = 0.017, failure load: *p* = 0.018; tibia: stiffness: *p* = 0.007, failure load: *p* = 0.007.). For vBMD, there was a statistically significant difference of the Tot.vBMD of the distal tibia among three groups (*p* = 0.015) and the highest tertile group of serum Nell‐1 level had highest Tot.vBMD. For cortical bone, we found that both the Ct.Ar and Ct.Th at the distal tibia were significantly different among three groups (Ct.Ar: *p* = 0.002, Ct.Th: *p* = 0.003). After analysis between two groups, there were larger Ct.Ar and thicker Ct.Th at the distal tibia in the highest tertile group than the second tertile group (Ct.Ar: *p* = 0.001, Ct.Th: *p* = 0.002). In bivariate correlation analysis, we also found that the Ct.Ar at the distal tibia was positively correlated with serum Nell‐1 level (*r* = 0.189, *p* = 0.036). For trabecular bone, the Tb.Th at the distal tibia was significantly different among three groups (*p* = 0.003). Further exploration, the lowest tertile group of serum Nell‐1 level had thicker Tb.Th at the distal tibia than the second tertile group (*p* = 0.003). Similarly, there was negative correlation between the Tb.Th of the distal tibia and serum Nell‐1 level (*r* = −0.196, *p* = 0.030). At the same time, we attempted to divide the patients into OP and non‐OP groups; however, no difference in serum Nell‐1 levels was observed between the two groups.

### 3.3. Association Between Serum Nell‐1 Level and Muscle Mass, Muscle Function, Risk of Falls, and Fractures

ASM obtained by DXA could be used to evaluate muscle mass. Maximum grip strength could be used to assess upper extremity function, and SPPB test could be used to assess lower extremity function in older adults. TUG test was also a simple and commonly used test to assess a person’s mobility, balance, walking ability, and risk of falling, especially in elderly or physically impaired individuals. Fracture depended not only on bone quality but also on muscle function, body balance ability, motor ability, and the risk of falling. Therefore, we tried to find the potential connections between serum Nell‐1 levels and the above indicators to explore whether Nell‐1 play a role in muscle, body control, and motor ability, with results presented in Table 3 and Supporting Table 3. There was no significant difference among the three groups in muscle mass, muscle function, risk of falls, and fractures. No correlation was found between the serum Nell‐1 level and indicators about the muscle mass, muscle function, risk of falls, and fractures. In further analysis, when the cohort was segmented based on the presence of sarcopenia, the risk of fall evaluated by the time of TUG test, or a history of falls and fractures, no significant disparities in serum Nell‐1 levels were identified. This consistency across groups suggests that Nell‐1 may not play a pivotal role in muscle physiology within the parameters measured in our study population.

**TABLE 3:** Muscle mass, muscle function, and the history of falls and fractures among postmenopausal women according to tertiles of serum Nell‐1 level.

	ALL (*N* = 123)	T1 (*n* = 41)	T2 (*n* = 41)	T3 (*n* = 41)	*P*	*P*_1_	*P*_2_	*P*_3_
Muscle mass								
ASM (kg)	14.73 (13.51, 16.44)	14.41 (13.10, 16.43)	14.62 (12.98, 16.27)	15.05 (13.96, 16.62)	0.298			
ASMI (kg/m^2^)	6.16 (5.69, 6.72)	6.19 (5.74, 6.92)	6.12 (5.59, 6.60)	6.16 (5.75, 6.77)	0.521			
Muscle function								
Maximum grip strength (kg)^a^	21.52 ± 4.60	20.68 ± 5.16	21.64 ± 4.41	22.23 ± 4.18	0.311	1.000	0.392	1.000
The score of the standing balance test^a^	4.0 (4.0, 4.0)	4.0 (4.0, 4.0)	4.0 (4.0, 4.0)	4.0 (4.0, 4.0)	0.321			
The score of the 2.44‐m gait speed test^a^	4.0 (4.0, 4.0)	4.0 (4.0, 4.0)	4.0 (4.0, 4.0)	4.0 (4.0, 4.0)	0.268			
The score of the 5‐time chair stand test^a^	4.0 (3.0, 4.0)	4.0 (3.0, 4.0)	4.0 (4.0, 4.0)	4.0 (3.5, 4.0)	0.530			
The score of the SPPB test^a^	12.0 (11.0, 12.0)	12.0 (11.0, 12.0)	12.0 (11.0, 12.0)	12.0 (10.0, 12.0)	0.365			
The time of the TUG test (s)^b^	8.13 (7.20, 10.05)	8.32 (7.37, 9.97)	8.30 (7.16, 10.25)	7.93 (7.11, 9.86)	0.807			
The risk of falls and the history of falls								
The history of falls in recent 1 year	25.2% (31/123)	29.3% (12/41)	19.5% (8/41)	26.8% (11/41)	0.571			
The history of fractures								
The history of fractures after age 50	29.3% (36/123)	29.3% (12/41)	26.8% (11/41)	31.7% (13/41)	0.889			
The history of fractures in recent 1 year^c^	3.3% (4/122)	2.4% (1/41)	2.5% (1/40)	4.9% (2/41)	0.779			

*Note:* All subjects were grouped according to tertiles of serum Nell‐1 level. Normally distributed continuous variables were depicted as mean ± standard deviation (SD) and non‐normally distributed continuous variables were shown as median (interquartile range, IQR). Categorical variables were expressed as proportion (counts/sum). Normally distributed continuous variables were analyzed by the one‐way analysis of variance (ANOVA). Non‐normally distributed continuous variables were analyzed by the Kruskal–Wallis H test. The classified data were analyzed by the Chi‐square test or Fisher’s exact test. *p* values were adjusted using the Bonferroni correction method to control for Type I error. The original significance level was 0.05, and the adjusted significance level was 0.05/3 ≈ 0.0167. *P*_1_ represents the comparison between the T1 and T2 groups; *P*_2_ represents the comparison between the T1 and T3groups; *P*_3_ represents the comparison between the T2 and T3 groups. ASM, appendicular skeletal muscle mass; ASMI, appendicular skeletal muscle mass index; TUG, Timed Up and Go.

Abbreviation: SPPB, Short Physical Performance Battery.

^a^ *N* = 122, T1 *n* = 40, T2 *n* = 41, T3 *n* = 41.

^b^ *N* = 121, T1 *n* = 40, T2 *n* = 40, T3 *n* = 41.

^c^ *N* = 122, T1 *n* = 41, T2 *n* = 40, T3 *n* = 41.

## 4. Discussion

This study represented the first exploration of the relationship between serum Nell‐1 level and various parameters associated with OP in a cohort of postmenopausal Chinese women, including BMD, bone microarchitecture, muscle mass, fall risk, and fracture incidence. Our findings revealed that postmenopausal women with elevated serum Nell‐1 level showed both higher femoral neck aBMD and total hip aBMD. Additionally, those with higher serum Nell‐1 level exhibited increased Tot.vBMD, larger Ct.Ar, thicker Ct.Th, as well as enhanced stiffness and failure load of the distal tibia. Notably, multiple linear regression analysis demonstrated a significant positive correlation between serum Nell‐1 levels and the Ct.Ar of the tibia after adjusting for various confounders. Interestingly, our findings suggested that Nell‐1 may be more closely associated with weight‐bearing cortical bone, while no significant correlations were observed between serum Nell‐1 level and the history of fracture, muscle mass, or muscle function. Our findings still highlight the positive impact of serum Nell‐1 in enhancing bone microstructure and strength. This positions Nell‐1 as a promising independent predictor for the assessment of bone health.

In our study, we identified a positive correlation between elevated serum Nell‐1 levels and increased aBMD at the femoral neck and total hip in postmenopausal women, a finding consistent with prior animal research. Specifically, administration of Nell‐1‐PEG led to a progressive and statistically significant enhancement in aBMD at the distal femurs 4 weeks following treatment, as compared to baseline measurements [19]. Furthermore, in osteoporotic sheep, targeted rhNell‐1 administration resulted in a marked increase in aBMD of the lumbar vertebrae [14]. Notably, our research exclusively revealed an association between serum Nell‐1 levels and aBMD at the femoral neck and total hip, with no such link detected between serum Nell‐1 and aBMD at L1‐4. This may be due to the fact that some patients in this cohort had experienced lumbar vertebral compression fractures, which may have prevented aBMD of L1‐4 from accurately reflecting the true bone health of these patients.

We further identified a positive correlation between elevated serum Nell‐1 levels and enhanced bone quality, as evidenced by improvements in bone microarchitecture and biomechanical properties. Specifically, individuals with higher serum Nell‐1 levels demonstrated increased Tot.vBMD at the distal tibia, along with augmented Ct.Ar and Ct.Th. These observations are supported by animal studies; for instance, in a study involving ovariectomy‐induced osteoporotic senile rats, the Nell‐1‐treated group exhibited a significantly increase in Tot.vBMD at the proximal and mid‐shaft of the femur compared to control group [20]. Additionally, histological and histomorphometric analyses of osteoporotic sheep’s lumbar vertebrae showed a dose‐dependent and significant increase in cortical width in the rhNell‐1‐treated group relative to the control group [14]. More importantly, our study revealed that participants with higher Nell‐1 level had enhanced stiffness and failure load at the distal tibia. Consistent with these findings, a study in rabbits that underwent tibial osteotomy and distraction found that the rhNell‐1 group presented higher peak load of the lengthened tibia than the saline group [21], and another study indicated that the rhNell‐1‐treated group displayed a more stress‐resistant composition in the lumbar vertebrae of osteoporotic sheep compared to the control group [14]. Collectively, these findings substantiate the association between higher Nell‐1 levels and better bone quality.

As for fractures history, no association was identified between serum Nell‐1 levels and fracture incidence in postmenopausal women. This finding is not in conflict with studies on open fracture animal models, which reported enhanced healing and increased callus formation with Nell‐1 treatment [21]. The differences may stem from diverse factors, and we hypothesize that Nell‐1 might be predominantly engaged in the fracture healing process. Our study, being retrospective, may not accurately reveal the acute state as it primarily involves patients with healed fractures.

More interestingly, we observed that Nell‐1 levels were positively correlated with the height of the subjects. This finding provides a novel perspective for understanding the potential role of Nell‐1 in skeletal development. A previous study suggested that Nell‐1 was a functional modulator of chondrogenesis [10]. We hypothesize that the correlation between Nell‐1 levels and height may be related to its role in the growth plate of bones, particularly in the proliferation, differentiation, and eventual ossification of chondrocytes. However, this hypothesis still needs to be validated through further experimental research to elucidate the precise mechanisms by which Nell‐1 regulates skeletal growth. Furthermore, we detected a negative correlation between serum Nell‐1 levels and β‐CTX, with no significant correlations with other bone turnover markers. Prior research indicates that Nell‐1 promotes the differentiation of osteoblast precursors and suppresses osteoclast precursor differentiation [19, 20], which could partially account for our findings. However, the influence of anti‐osteoporotic medications used by some participants on bone turnover markers cannot be discounted. Additionally, human bone turnover markers may be influenced by a multitude of confounding factors not present in animal experiments.

In our attempt to elucidate the relationship between serum Nell‐1 levels and muscular health, we observed no significant correlations between serum Nell‐1 levels and either muscle mass or muscle function, indicating a potentially limited role of Nell‐1 in muscular health. Additionally, our research did not reveal any significant associations between serum Nell‐1 levels and the risk of falls. Fall risk is inherently multifactorial, with key determinants being the maintenance of balance and gait stability, which are highly dependent on competent muscular control. Collectively, these findings suggest that in the cohort of postmenopausal women, Nell‐1 may be more intricately associated with skeletal health rather than muscular health.

This study represents the first attempt within a clinical cohort to investigate the relationship between serum Nell‐1 protein levels and skeletal health in postmenopausal women. Although our research has provided some insights, it encounters several constraints. The cross‐sectional design limits our ability to further explore the deeper mechanisms behind these findings. Second, the enrollment of a mere 24% osteoporotic patients and a low incidence of fractures among our participants restrict the in‐depth exploration of the role of Nell‐1 in the prevention and treatment of OP. Moreover, to clarify the potential of serum Nell‐1 levels as a biomarker for skeletal system function in postmenopausal women, future studies will require an increased number of participants with OP.

In conclusion, our study indicates that postmenopausal women with higher Nell‐1 level had higher hip aBMD, higher Tot.vBMD, and higher estimated bone strength. These results underscore the potential significance of serum Nell‐1 levels in relation to skeletal health, providing population‐based evidence that Nell‐1 could be a potential therapeutic target for OP.

## Author Contributions

All authors were involved in drafting the article or revising it critically for important intellectual content. Experimental work, original draft preparations, and methodology: Yiyi Gong, Yushuo Wu, and Xiang Li; study conception and design: Weibo Xia and Yan Jiang; acquisition of data: Xiaosen Ma, Xiaolin Ni, Wei Liu, Lijia Cui, Yue Chi, Ruizhi Jiajue, and Qianqian Pang; analysis and interpretation of data: Dr. Yan Jiang, Dr. Ou Wang, Dr. Mei Li, Dr. Zaizhu Zhang, and Dr. Xiaoping Xing.

## Funding

This study was supported by the National Key R&D Program of China (2021YFC2501700), National Natural Science Fund (No. 82100942), CAMS Innovation Fund for Medical Sciences (CIFMS) (2021‐I2M‐1‐002), and National High Level Hospital Clinical Research Funding (2022‐PUMCH‐D‐006).

## Disclosure

All authors approved the final version to be published.

## Ethics Statement

The study was approved by the ethical committee of Peking Union Medical College Hospital (reference number: JS‐2905).

## Conflicts of Interest

The authors declare no conflicts of interest.

## Supporting Information

Concise description of Supporting information.

Supporting Table 1. Correlation analysis between serum Nell‐1 level and general characteristics and biochemical parameters.

Supporting Table 2. Correlation analysis between serum Nell‐1 level and BMD, lumbar TBS, and HR‐pQCT parameters.

Supporting Table 3. Correlation analysis between serum Nell‐1 level and muscle mass, muscle function, and the history of falls and fractures.

## Supporting information


**Supporting Information** Additional supporting information can be found online in the Supporting Information section.

## Data Availability

The data that support the findings of this study are available from the corresponding author upon reasonable request.
