# Supplementary material for: Relationship Between Serum Nell‐1 Level and Bone Geometry, Bone Microarchitecture in Chinese Postmenopausal Women
Source: Int J Endocrinol. 2026 Jan 31;2026:9977862. doi: 10.1155/ije/9977862 (PMC12860418; doi:10.1155/ije/9977862)
Supplement: Supplementary file 1 — Supporting Information Additional supporting information can be found online in the Supporting Information section. [file IJE-2026-9977862-s001.docx]

**Supplement Table 1. Correlation analysis between serum Nell**-**1 level and general characteristics and biochemical parameters**

|  |  | **r** | ***P*** |
| --- | --- | --- | --- |
| Age (y) | 69.0 (64.0, 78.0) | **-**0.138 | 0.125 |
| Height (cm) | 155.3 ± 6.8 | **0.206** | **0.022** |
| Weight (kg) | 60.0 (54.0, 68.0) | 0.151 | 0.096 |
| BMI (kg/m^2^) | 25.57 ± 4.05 | 0.069 | 0.452 |
| Ca (mmol/L) | 2.35 ± 0.09 | 0.039 | 0.670 |
| Pi (mmol/L) | 1.18 (1.07, 1.31) | **-**0.117 | 0.199 |
| ALP (U/L) | 79.0 (71.0, 91.0) | **-**0.055 | 0.535 |
| iPTH (pg/ml) | 48.6 (36.8, 63.4) | **-**0.004 | 0.962 |
| 25(OH)D (ng/ml) | 21.6 (16.4, 27.1) | 0.049 | 0.593 |
| β-CTX (ng/ml) | 0.40 (0.28, 0.49) | **-0.183** | **0.043** |
| P1NP (ng/ml) | 45.7 (35.0, 57.7) | **-**0.130 | 0.151 |
| Cr (µmol/L) | 64.0 (58.0, 77.0) | **-**0.152 | 0.093 |

Normally distributed continuous variables were depicted as mean ± standard deviation (SD) and non-normally distributed continuous variables were shown as median (interquartile range, IQR). Spearman analysis was used between serum Nell-1 level and general characteristics and biochemical parameters. Bold values denoted statistically significant differences (*P* < 0.05).

Abbreviations: BMI, body mass index; Ca, serum total calcium; Pi, serum phosphate; ALP, serum alkaline phosphatase; iPTH, serum intact parathyroid hormone; 25(OH)D, 25**-**hydroxy vitamin D; β**-**CTX, C**-**terminal cross-linking telopeptide of type I collagen; P1NP, procollagen type 1 N-terminal pro-peptide; Cr, serum creatinine.

**Supplement Table 2. Correlation analysis between serum Nell**-**1 level and BMD, lumbar TBS and HR**-**pQCT parameters**

| **aBMD** | | | | | | |
| --- | --- | --- | --- | --- | --- | --- |
|  | **BMD** | | | **T-score** | | |
|  |  | **r** | ***P*** |  | **r** | ***P*** |
| L1**-**4 | 1.056 ± 0.183 | 0.067 | 0.467 | **-**0.56 ± 1.48 | 0.064 | 0.481 |
| Femoral neck | 0.774 ± 0.129 | 0.159 | 0.078 | **-**1.16 ± 1.08 | 0.136 | 0.133 |
| Total hip | 0.843 ± 0.146 | 0.100 | 0.271 | **-**0.89 ± 1.17 | 0.047 | 0.602 |
| **lumbar TBS** | | | | | | |
|  |  | **r** | | ***P*** | | |
|  | 1.260 ± 0.096 | 0.079 | | 0.391 | | |
| **HR**-**pQCT parameters** | | | | | | |
|  | **Radius** | | | **Tibia** | | |
|  |  | **r** | ***P*** |  | **r** | ***P*** |
| **Bone geometry** | | | | | | |
| Tot.Ar (mm^2^) | 248.5 ± 39.6 | 0.021 | 0.817 | 651.5 ± 112.5 | 0.083 | 0.359 |
| Tb.Ar (mm^2^) | 199.7 ± 40.6 | **-**0.010 | 0.909 | 559.5 ± 108.9 | 0.039 | 0.666 |
| Ct.Ar (mm^2^) | 52.3 ± 10.4 | 0.134 | 0.140 | **100.7 ± 19.9** | **0.189** | **0.036** |
| Ct.Pm (mm) | 65.9 ± 6.1 | 0.019 | 0.836 | 99.6 ± 8.2 | 0.101 | 0.265 |
| **vBMD** | | | | | | |
| Tot.vBMD (mgHA/cm^3^) | 256.8 (211.9, 301.6) | 0.076 | 0.401 | 221.9 (187.9, 268.2) | 0.039 | 0.672 |
| Tb.vBMD (mgHA/cm^3^) | 91.5 (71.7, 122.6) | 0.051 | 0.573 | 116.3 ± 38.4 | **-**0.040 | 0.658 |
| Ct.vBMD (mgHA/cm^3^) | 892.5 ± 70.5 | 0.074 | 0.415 | 842.3 ± 65.1 | 0.102 | 0.263 |
| **Bone microarchitecture** | | | | | | |
| Tb.N (1/mm) | 1.123 ± 0.306 | 0.065 | 0.474 | 1.125 (0.972, 1.249) | 0.022 | 0.805 |
| Tb.Th (mm) | 0.218 (0.210, 0.229) | **-**0.072 | 0.428 | **0.246 ± 0.020** | -**0.196** | **0.030** |
| Tb.Sp (mm) | 0.862 (0.737, 1.096) | **-**0.065 | 0.475 | 0.872 (0.783, 1.038) | **-**0.036 | 0.696 |
| Ct.Th (mm) | 0.950 (0.794, 1.049) | 0.118 | 0.193 | 1.216 ± 0.258 | 0.110 | 0.224 |
| Ct.Po | 0.007 (0.004, 0.010) | 0.025 | 0.786 | 0.033 (0.023, 0.044) | **-**0.057 | 0.532 |
| **Estimated bone strength** | | | | | | |
| Stiffness  (N/mm) | 44894.9 ±  11542.9 | 0.096 | 0.290 | 130107.9 ±  30484.5 | 0.046 | 0.617 |
| Failure load (N) | 2386.6 ± 645.2 | 0.084 | 0.355 | 7123.2 ± 1625.7 | **-**0.059 | 0.521 |

Normally distributed continuous variables were depicted as mean ± standard deviation (SD) and non-normally distributed continuous variables were shown as median (interquartile range, IQR). Spearman analysis was used between serum Nell-1 level and BMD, lumbar TBS and HR-pQCT parameters. *P_1_* adjusted nothing*,* *P_2_* adjusted by age*, P_3_* adjusted by age, height, weight were the results from linear regression analysis between serum Nell-1 level and BMD, lumbar TBS and HR-pQCT parameters. Bold values denoted statistically significant differences (*P* < 0.05).

Abbreviations: aBMD, areal bone mineral density; L1-4, lumbar vertebrae 1-4; TBS, trabecular bone score; HR-pQCT, high-resolution peripheral quantitative computed tomography; Tot.Ar, total area; Tb.Ar, trabecular area; Ct.Ar, cortical area; Ct.Pm, cortical perimeter; vBMD, volumetric bonemineral density; Tot.vBMD, total vBMD; Tb.vBMD, trabecular vBMD; Ct.vBMD, cortical vBMD; Tb.N, trabecular number; Tb.Th, trabecular thickness; Tb.Sp, trabecular separation; Ct.Th, cortical thickness; Ct.Po, cortical porosity.

**Supplement Table 3. Correlation analysis between serum Nell**-**1 level and muscle mass, muscle function and the history of falls and fractures**

|  |  | **r** | ***P*** |
| --- | --- | --- | --- |
| **Muscle mass** | | | |
| ASM (kg) | 14.73 (13.51, 16.44) | 0.134 | 0.139 |
| ASMI (kg/m^2^) | 6.16 (5.69, 6.72) | 0.036 | 0.690 |
| **Muscle function** | | | |
| Maximum grip strength (kg) | 21.52 ± 4.60 | 0.173 | 0.057 |
| The score of the standing balance test | 4.0 (4.0, 4.0) | 0.012 | 0.898 |
| The score of the 2.44**-**meter gait speed test | 4.0 (4.0, 4.0) | **-**0.036 | 0.695 |
| The score of the 5**-**time chair stand test | 4.0 (3.0, 4.0) | 0.063 | 0.488 |
| The score of the SPPB test | 12.0 (11.0, 12.0) | 0.048 | 0.602 |
| The time of the TUG test (s) | 8.13 (7.20, 10.05) | **-**0.056 | 0.540 |
| **The history of falls in recent one year** | 25.2% (31/123) | **-**0.091 | 0.221 |
| **The history of fractures after age 50** | 29.3% (36/123) | 0.013 | 0.861 |
| **The history of fractures in recent one year** | 3.3% (4/122) | **-**0.002 | 0.983 |

Normally distributed continuous variables were depicted as mean ± standard deviation (SD) and non-normally distributed continuous variables were shown as median (interquartile range, IQR). Categorical variables were expressed as proportion (counts/sum). Spearman analysis and Kendall’s tau-b analysis were used between serum Nell-1 level and muscle mass, muscle function and the history of falls and fractures. Bold values denoted statistically significant differences (*P* < 0.05).

Abbreviations: ASM, appendicular skeletal muscle mass; ASMI, appendicular skeletal muscle mass index; SPPB, Short Physical Performance Battery; TUG, Timed Up and Go.
